# Quantitative electroencephalography reveals different physiological profiles between benign and remitting-relapsing multiple sclerosis patients

**DOI:** 10.1186/1471-2377-8-44

**Published:** 2008-11-24

**Authors:** Manuel Vazquez-Marrufo, Javier J Gonzalez-Rosa, Encarnacion Vaquero, Pablo Duque, Monica Borges, Carlos Gomez, Guillermo Izquierdo

**Affiliations:** 1 Laboratory of Psychophysiology, Department of Experimental Psychology, University of Seville, Camilo Jose Cela s/n, 41018 Seville, Spain; 2 Multiple Sclerosis Unit, Virgen Macarena Hospital, Avda Dr Fedriani s/n, 41009 Seville, Spain

## Abstract

**Background:**

A possible method of finding physiological markers of multiple sclerosis (MS) is the application of EEG quantification (QEEG) of brain activity when the subject is stressed by the demands of a cognitive task. In particular, modulations of the spectral content that take place in the EEG of patients with multiple sclerosis remitting-relapsing (RRMS) and benign multiple sclerosis (BMS) during a visuo-spatial task need to be observed.

**Methods:**

The sample consisted of 19 patients with RRMS, 10 with BMS, and 21 control subjects. All patients were free of medication and had not relapsed within the last month. The power spectral density (PSD) of different EEG bands was calculated by Fast-Fourier-Transformation (FFT), those analysed being delta, theta, alpha, beta and gamma. Z-transformation was performed to observe individual profiles in each experimental group for spectral modulations. Lastly, correlation analyses was performed between QEEG values and other variables from participants in the study (age, EDSS, years of evolution and cognitive performance).

**Results:**

Nearly half (42%) the RRMS patients showed a statistically significant increase of two or more standard deviations (SD) compared to the control mean value for the beta-2 and gamma bands (F = 2.074, p = 0.004). These alterations were localized to the anterior regions of the right hemisphere, and bilaterally to the posterior areas of the scalp. None of the BMS patients or control subjects had values outside the range of ± 2 SD. There were no significant correlations between these values and the other variables analysed (age, EDSS, years of evolution or behavioural performance).

**Conclusion:**

During the attentional processing, changes in the high EEG spectrum (beta-2 and gamma) in MS patients exhibit physiological alterations that are not normally detected by spontaneous EEG analysis. The different spectral pattern between pathological and controls groups could represent specific changes for the RRMS patients, indicative of compensatory mechanisms or cortical excitatory states representative of some phases during the RRMS course that are not present in the BMS group.

## Background

Multiple sclerosis (MS) is generally regarded as a chronic inflammatory demyelinating condition leading to focal demyelination plaques in white matter, although recent studies have demonstrated the presence of lesions in the cerebral cortex and brain stem nuclei [1,2]. Axons can be damaged either within inflammatory lesions [3] or, at a later stage, in chronically demyelinated plaques due to the lack of trophic support [4].

Along with the pathophysiology, it has been estimated that cognitive impairment in MS occurs in 40–70% of cases, probably resulting from disruption of cortical and subcortical pathways as a consequence of demyelation and axonal transection [5,6]. Nevertheless, great variability exists in cognitive performance of individual MS patients, implying that cognitive preservation and deterioration occurs during the evolution of the disease [7,8].

Different forms of MS vary widely in their typical clinical course [9-11]. In most patients with MS, clinical onset is characterized by relapses and remissions, with episodes of neurological impairment. This typical clinical presentation in relapsing-remitting multiple sclerosis (RRMS) accounts for >80% of cases. Most of these patients inevitably progress towards disability (secondary progressive multiple sclerosis, SPMS). Another group of patients do not have relapses at the onset of MS, but steadily accumulate disability over time (primary progressive multiple sclerosis, PPMS) [12,13].

MS is characterized by a great heterogeneity in its long-term prognosis and the distinctions between these typical clinical phenotypes are not absolute. Taking SPMS as an example, the time to conversion, the rate of progression and the relative contribution of relapses and progression may vary dramatically. In some patients, a 'benign' clinical course (BMS) is also observed. Although the definition is arbitrary, the prevalence of BMS relates to 10–20% of patients whose EDSS score remains below 3 or 3.5 (mild disability) after at least 10 or 15 years from the disease onset [14-17].

One of the challenges of MS is to distinguish different physiological alterations among the diverse clinical subtypes [18]. In particular, it is desirable to find alterations that start from the early beginning of the disease, which would allow a fast and accurate diagnostic classification of the patient and an easier decision about its management [19].

The most relevant paraclinical technique used in the early evaluation of MS is probably MRI [20]. However, MRI gives a little specificity in differentiating between MS groups, although new approaches (fMRI or Diffusion Tensor Imaging, DTI) seem promising for future investigations [21-23].

Due to the fact that MS is a demyelination disease, the lack of myelin alters the physiological activity of neurons in the central nervous system (CNS). One means of analysing this alteration is the electroencephalogram (EEG) that records mainly the neural activity of cortical neurons. Several studies have shown abnormal brain activity related to neurological or psychiatric diseases [24-26].

In the case of MS, different studies have looked at possible relationships between EEG activity and different aspects of the MS disease. One study [27] observed changes in the beta activity in fronto-central areas of the scalp which were directly correlated with the disability score (the higher disability score, the higher beta activity). Another study [28] determined whether EEG could detect a possible association between epilepsy and MS, but it failed to provide a satisfactory estimate of presence of abnormal brain activity in MS patients. In the case of alterations of the quantitative spectral content of the EEG (QEEG) in MS, a common result is the heterogeneity of modulations displayed in these patients along all bands [28,29]. Moreover, the correlation between spectral scores and the degree of cognitive impairment assessed by neuropsychological testing was low in most cases [27,30]. In the latter study, the cognitive status of a group of elderly subjects indexed by the Mini Mental State Examination (MMSE) was not significantly correlated with QEEG scores. For MS, another study [27] showed that QEEG did not give as valid an estimation of neurological parameters as SNE (standard neurological examination).

Therefore, the general impression of QEEG in MS is that it is of little use as a technique, but might support diagnostic reports provided by neurological and/or MRI exploration [31,32]. However, some experts in the QEEG field believe it is possible to correlate different patterns in EEG spectra with particular pathological conditions [24].

A new approach is needed to reveal specific patterns in MS disease. Usually, the quantification of the EEG spectrum is based in the application of Fast Fourier Transformation (FFT) in concrete intervals under a passive condition of the subject. But an alternative is to apply the same protocol during intervals when the subject is performing a cognitive task that stresses areas that may have been afflicted by the disease. One such study [33] showed an increment in the high bands of the EEG spectrum (beta and gamma) during an auditory oddball task in RRMS patients. The modulations were present in the frontal areas of the scalp and might have represented a cognitive impairment related to automatic reorientation mechanisms of the auditory attentional system.

With evidence of specific patterns of QEEG in different neuropathologies and the knowledge that abnormal brain activity in MS patients can occur when a cognitive task provokes a stress condition, the main question was posed as to whether it would it be possible to distinguish different physiological profiles in diverse groups of MS patients? Of particularly interest would be to find specific physiological markers that distinguish between the BMS and RRMS groups because an early diagnosis of the type of MS could greatly help in deciding therapy and future management.

Hence, our principal aim is to report possible QEEG alterations in RRMS and BMS patients during the execution of an attentional task that involves many areas in the brain, i.e. in all probability, those that are probably impaired by their pathological condition. In particular, we have analysed the QEEG and behavioural performance elicited during a visuo-spatial task (Posner paradigm), which consists of trials with central cues indicating the most likely position of a subsequent target that has to be discriminated by the subject. In some cases, the cue indicates correctly the future location of the target ("valid trials"), whereas when the cue directs attention to the opposite area where the target will be presented, these trials are defined as "invalid". Typically, longer reaction times were found for invalid trials compared to valid ones [34]. The comparison between valid and invalid trials shows that it is possible to study the fixation of our spatial attention in the visual field and the cost related in shifting the spatial attention to another area [34,35].

The specific objectives in the study were the following: 1) to check if there are diverse patterns of spectral content for different groups of patients compared to a normal group matched in age, gender and educational status (in the affirmative case, the present study seeks to test if these differences are consistent through all subjects or are present in some of them but not all): 2) to compare the results obtained with a visual modality with those of a previous study [33] using an auditory attentional paradigm (the major concern are the bands that are altered and the topography exhibited by them): and 3) to carry out a correlation analysis between QEEG scores and several scores (neurological and psychophysiological measures).

## Methods

### Subjects and procedure

Three different groups of subjects participated in the study: The first group included 19 patients (women 14; age 36.95 ± 7.71 years old) diagnosed with RRMS without clear signs of motor impairment, and with disease duration of 5.42 ± 4.21 years. The score in Multiple Sclerosis Expanded Disability Status Scale (EDSS) for this group was always equal or fewer than 3.5 (mean: 1.58 ± 0.8). The second group included 10 patients (women 7; age 40.50 ± 7.60 years old) diagnosed of BMS. These patients suffered MS during 12.30 ± 4.22 years and at least 8 years of disease evolution and scoring equal or fewer than 3.5 in the EDSS scale (mean: 2,00 ± 1.1). The data from both clinical groups were compared with a group of 21 healthy subjects (women 13; age 35.57 ± 7.82 years old) similar in age, gender proportion and educational level. The experimental protocol was approved by the ethical committee of the Hospital. After a full explanation about the details of the experiment, a written consent statement was obtained from MS patients and controls.

All patients participating in the study were classified using the Poser criteria [9]. We include in the experiment only subjects that were clinically stable at baseline (meaning no exacerbations within several months before the participation in the experiment, no medication at the time of evaluation (at least one-month window and only a few presented history of corticoids treatment in past relapses) and no signs of depression. Patients were previously assessed at the Multiple Sclerosis Unit of the Neurology Service of the Virgen Macarena Hospital (Seville, Spain) and participated voluntarily in the Psychophysiological testing.

### Experimental protocol

**Figure 1:**
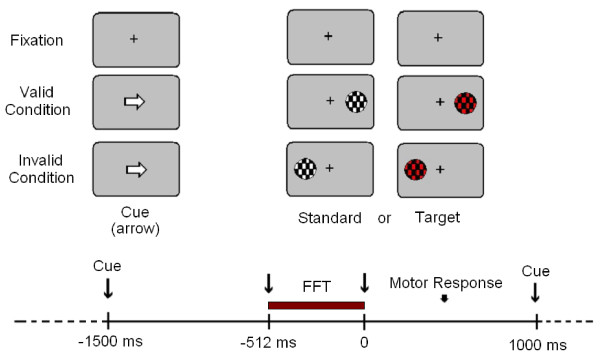
**Experimental Paradigm**. The onset of the imperative stimulus is on 0 milliseconds (ms). FFT: Fast Fourier Transformation Interval.

Behavioural responses were recorded during the Posner paradigm. Five blocks with 200 trials each were presented. There were pauses between blocks to prevent the appearance of fatigue in the subjects. Each trial consisted in a central cue (lasting 300 ms) pointing to the left or to the right side of the screen, where a target (mandatory response) or a standard stimulus (no response) appears. Targets and standards also lasted 300 ms. The sequence of cue and imperative stimulus lasted for 1.5 sec and the inter-trial time was 1 sec. The cue (a white arrow) appeared at the screen centre. In standard and target trials the cue could point to the position where the stimulus appears (valid trials, 80%) or to the opposite side (invalid trials, 20%) (see Figure 1). The shape of the target stimuli was a circle with a pattern of black and red checkerboard subtending a visual angle of 2.46 degrees. The standards presented the same shape as targets but the colours were changed to black and white. Both stimuli appeared randomly at left or right side in the visual field. Subjects had to press the left button of the mouse with their dominant hand when targets appeared in the left side, and the right button when the target appeared on the right side. Therefore, standard and target stimuli, depending on the cue and the position in the visual field, could be left valid, left invalid, right valid and right invalid. The paradigm used is a modified version of Posner's paradigm [35] with a 75% of standard stimuli.

The EEG was recorded from 13 electrodes (Fz, Cz, Pz, F3, F4, C3, F4, P3, F4, T5, F6, O1, O2) from the 10–20 International System [36]. The electrodes were referenced to the left mastoid and re-referenced off-line to the right mastoid. Data were filtered using a band-pass of 0.01–100 Hz (1/2 amplitude low- and high-frequency cut-offs); the amplification gain was 20,000. The EOG was recorded with bipolar recording by means of electrodes situated in the external canthi of the ocular orbits and in the inferior and superior positions of the left orbit. The impedance was kept under 5 kΩ. Artefacts were automatically detected and visually revised. The trials in which HEOG artefacts higher than ± 70 μV were detected at electrodes Fz, Cz or Pz, were rejected. The baseline was 100 ms prior to the standard and target stimuli. The different experimental conditions were averaged independently.

### EEG analysis

The power spectral density for each band (PSD) was calculated by means of the Fast-Fourier Transformation [37]. The selected time window for the analysis was from 512 ms previous to the onset of the imperative stimulus until this onset (spectral resolution: 2 Hz). The digitisation rate used for the EEG recording was of 500 Hz/channel that is enough according to the Nyquist criteria to carry out an appropriate analysis of all the frequencies of our study [38]. The analysed bands were delta (0.5. 4 Hz), theta (5–8 Hz), alpha (9–12 Hz), beta 1 (13–21 Hz) beta 2 (22–30) gamma and (31–45 Hz). To avoid the leakage effect, a cosine window was applied on the borders of the time segment for analysis [38]. Logarithmic transformation was also applied for any PSD value to achieve a valid normal distribution of these data and allow an ANOVA analysis [39].

The same analysis was performed for the background EEG activity occurring during the pause blocks of the experiment to test if the possible spectral modulations were present even in the absence of the execution of the cognitive task.

### Normalization procedure

A normalization procedure was applied to the QEEG data and behavioural measures to analyse the individual scores of every subject from RRMS, BMS and control groups. The purpose of this analysis is to observe whether heterogeneity or homogeneity is present in the abnormal PSD values and behavioural measures along MS patients. To perform this procedure the control group was taken as the normative sample (mean and standard deviation) and the Z-Transformation was calculated following the formula:

Z = (xi - X)/SD

Where xi is the PSD value for every patient in different scalp derivations, X and SD were mean and standard deviations calculated from the control group respectively. An average of Z-values was calculated for each subject across all scalp derivations that were different between RRMS and the rest of the groups defined by the post-hoc analysis performed for the PSD scores. In the present study these derivations were F4, O1 and O2.

### Statistical analysis

#### Behavioural analysis

The behavioural parameters analysed were reaction time (RT) to the target stimuli and percentage of correct responses (CR). An ANOVA design for repeated measurements was used to analyse the RTs and CRs data. The intra-subject factor was the validity of the cue (two levels: valid vs. invalid) and the intersubject factor was the subject's group (three levels: RRMS, BMS and Control group).

#### QEEG analysis

An ANOVA design for repeated measures was used to analyse the spectral modulations. The intra-subject factors were Spectral Band (delta, theta, alpha, beta-1, beta-2 and gamma); Stimulus location (left and right); Hemisphere (left and right); Electrode position (frontal, central, parietal, occipital). Inter-subjects factor: Experimental group: Control, RRMS and BMS). Greenhouse-Geisser correction for sphericity was applied. Bonferroni correction was carried out in multiple comparisons post-hoc analysis.

#### Correlation analysis

Correlations between subject data (age), clinical parameters (EDSS or duration of disease), behavioural measures (reaction time and percentage of correct responses) and QEEG values were computed using Pearson's correlation coefficient. Data was considered significant at the 0.05 level.

## Results

### Reaction time and percentage of correct responses

The first relevant result in the analysis of the behavioural responses was the presence of the validity effect, i.e. of faster responses for the valid condition compared to the invalid ones (RRMS group (t = 4,319, p < 0.001) (18 ms) and the control group (t = 9.773, p < 0.001) (33 ms)). In the BMS group, the difference between the two conditions was not statistically significant (12 ms) (see table 1).

**Table 1:** Descriptive and Statistical results of the behavioural data

	**Reaction Time**			**Correct Responses %**		
**Descriptive**	**Valid**	**Invalid**	**Total**	**Valid**	**Invalid**	**Total**
BMS	545 ± 73	557 ± 65	551 ± 67	81 ± 24	80 ± 21	81 ± 22
RRMS	507 ± 57	525 ± 57	516 ± 56	89 ± 15	88 ± 15	89 ± 15
CON	441 ± 62	474 ± 66	457 ± 64	97 ± 3	96 ± 6	96 ± 4
						
**Anova**	**F**		***p***	**F**		***P***
Cue	43.007		<0.001	0.275		0.603
Group	8.891		0.001	4.627		0.015
Cue × Group	3.773		0.030	0.013		0.987
						
**Post Hoc**	**RRMS**	**BMS**	**CON**	**RRMS**	**BMS**	**CON**
RRMS		0.403	**0.006**		0.433	0.307
BMS			**<0.001**			**0.018**

CON: Control group; RRMS: relapsing-remitting group; BMS: Benign group.

Considering global responses, control subjects were faster than both MS groups (F [2,47] = 8.891, p = 0.001) (CON: 457 ms ± 64; RRMS: 516 ms ± 56; BMS: 551 ms ± 67) (see table 1). Moreover, ANOVA analysis revealed that an interaction effect took place between these two factors " cue × group" (F [2,47] = 8.990, p < 0.001). This result links the differences between groups observed by the main effect (the two MS groups have slower reaction times than control group); and on the other hand, an effect of validity exists in the RRMS and control groups that is not found in the BMS group.

Regarding the percentage of correct responses, no differences were found among the valid and invalid conditions of any of the groups. However, differences were observed among the different experimental groups (F [2,47] = 4.627, p = 0.015). Post-hoc analysis showed that BMS patients made significantly more errors than the control and RRMS groups (p = 0.018) (CON: 96 ms ± 4; RRMS: 89 ms ± 15; BMS: 81 ms ± 22) (see table 1).

### Quantitative EEG

**Figure 2:**
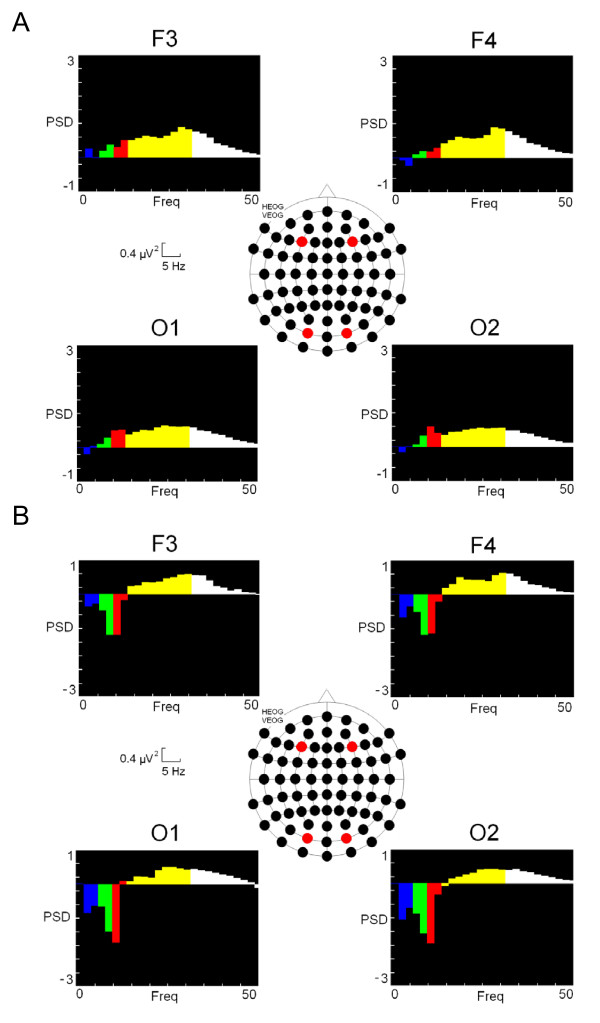
**Power Spectral Density (PSD) of different bands**. Different colours represent every spectral band (delta (0.5 – 4 Hz): blue colour; theta (5 – 8): green; alpha (9 – 12 Hz): red; beta (13 – 30 Hz): yellow and gamma (31 – 45 Hz): white. A) Subtraction of RRMS scores from control values. B) Subtraction of BMS scores from control values. CON: Control group; RRMS: relapsing-remitting group; BMS: Benign group.

In the spectral analysis, ANOVA analysis showed interactions between the following variables "Spectral Band × Stimulus Location × Hemisphere × Electrode × Group" with a probability of (F [2,47] = 1.941, p = 0.009). Post-hoc analysis indicated that the RRMS group was statistically significantly increased compared to the control group for the high bands of the spectrum (beta-2 (22–30 Hz) and gamma (31–45 Hz), in both the occipital regions (bilateral) (O1 and O2) and the frontal right hemisphere region (F4) (see figure 2 and table 2). No differences were found between the BMS and the control groups. The other bands investigated showed no significant differences. With respect to the background EEG, no differences in the high spectral bands were detected between the groups when subjects were not performing the attentional task.

**Table 2:** Post Hoc results for spectral analysis of beta-2 and gamma bands

	**BETA-2**							
**Descriptive**	**F3**		**F4**		**O1**		**O2**	
	
	**L**	**R**	**L**	**R**	**L**	**R**	**L**	**R**
BMS	2.152	2.185	1.986	1.984	1.445	1.467	1.419	1.418
RRMS	2.487	2.456	2.457	2.450	1.944	1.941	1.873	1.898
CON	1.850	1.877	1.748	1.770	1.307	1.320	1.306	1.307
								
**ANOVA**	**F**				***P***			
BxLxHxExG	1.941				0.009			

**Post Hoc**	**CON**		**CON**		**CON**		**CON**	
	**L**	**R**	**L**	**R**	**L**	**R**	**L**	**R**
RRMS	0.102	0.135	**0.022**	**0.040**	**0.023**	**0.035**	**0.037**	**0.042**
BMS	1.000	1.000	0.792	0.785	0.424	0.550	0.432	0.465

	**GAMMA**							

**Descriptive**	**F3**		**F4**		**O1**		**O2**	
	
	**L**	**R**	**L**	**R**	**L**	**R**	**L**	**R**
BMS	1.177	1.177	1.038	1.052	0.800	0.808	0.759	0.767
RRMS	1.325	1.290	1.300	1.289	1.094	1.085	1.059	1.040
CON	0.973	0.968	0.870	0.858	0.710	0.711	0.713	0.714
								
**ANOVA**	**F**				**P**			
BxLxHxExG	1.941				0.009			

**Post Hoc**	**CON**		**CON**		**CON**		**CON**	
	**L**	**R**	**L**	**R**	**L**	**R**	**L**	**R**
RRMS	0.196	0.182	**0.034**	**0.029**	**0.015**	**0.016**	**0.034**	**0.040**
BMS	1.000	1.000	0.945	1.000	0.435	0.517	0.348	0.434

CON: Control group; RRMS: relapsing-remitting group; BMS: Benign group. Side Stimulus Location: L (Left) and R (Right).

### Normalization

The results of Z-transformation of power spectral density values (PSD) for the averages of the F4, O1 and O2 derivations are given in table 3. Eight RRMS patients (out of 19) had PSD values for beta-2 and gamma of more than two standard deviations compared to the mean of the control. The same analysis for the BMS and the control groups showed that neither of them reached this limit.

**Table 3:** Clinical, behavioural and quantitative EEG data from subjects participating in the study

	**AGE**	**DE**	**EDSS**	**RT**	**BETA-2**	**GAMMA**
RRMS 1	32	4	2	587*	1.11	0.59
RRMS 2	31	2	0	488	2.37*	1.35*
RRMS 3	41	4	1.5	560	1.03	0.52
RRMS 4	29	2	1	540	1.51	0.84
RRMS 5	31	3	1.5	436	2.61*	1.44*
RRMS 6	52	8	3	455	2.76*	1.56*
RRMS 7	53	18	2	473	1.69	0.84
RRMS 8	42	2	2	548	1.06	0.59
RRMS 9	38	3	2.5	480	2.98*	1.88*
RRMS 10	35	5	1.5	411	1.3	0.71
RRMS 11	46	6	3	493	1.76	1.01
RRMS 12	32	3	1.5	552	1.02	0.53
RRMS 13	34	1	0	470	1.68	0.93
RRMS 14	41	5	1.5	535	4.87*	2.87*
RRMS 15	29	5	1	536	2.23	1.14
RRMS 16	34	6	1.5	547	2.55*	0.97
RRMS 17	40	14	1.5	587	2.06	1.1
RRMS 18	38	7	1.5	642*	2.46*	1.2
RRMS 19	24	5	1.5	552	2.67*	1.59*

BMS 1	45	10	1	552	1.91	1.09
BMS 2	37	8	0	606*	1	0.45
BMS 3	46	16	3.5	630*	1.31	0.71
BMS 4	56	11	2.5	616*	1.96	1.16
BMS 5	41	12	2	606*	1.59	0.81
BMS 6	37	14	2.5	500	1.7	0.77
BMS 7	36	22	2.5	418	1.11	0.69
BMS 8	28	12	1	490	1.95	0.95
BMS 9	43	8	3.5	544	1.93	1.06
BMS 10	36	10	1.5	544	1.7	0.97

CON 1	32	.	.	433	2.15	1.15
CON 2	31	.	.	406	1.88	0.9
CON 3	23	.	.	526	1.27	0.66
CON 4	35	.	.	489	2.01	1.11
CON 5	31	.	.	430	1.13	0.58
CON 6	54	.	.	476	1.09	0.56
CON 7	38	.	.	398	1.95	1.12
CON 8	33	.	.	507	1.5	0.81
CON 9	35	.	.	495	1.18	0.67
CON 10	51	.	.	484	1.47	0.68
CON 11	26	.	.	382	1.68	0.89
CON 12	36	.	.	342	1.81	0.94
CON 13	42	.	.	522	1.18	0.69
CON 14	35	.	.	505	1	0.5
CON 15	42	.	.	398	1.46	0.79
CON 16	36	.	.	422	1.09	0.58
CON 17	36	.	.	481	1.33	0.59
CON 18	35	.	.	419	1.35	0.67
CON 19	41	.	.	573	0.9	0.43
CON 20	34	.	.	550	1.73	0.89
CON 21	21	.	.	354	1.41	0.72

CON: Control group; RRMS: relapsing-remitting group; BMS: Benign group. DE: Disease evolution (years); EDSS: Expanded Disability Status Scale. RT: Reaction Time (ms); BETA-2 and GAMMA (Power Spectral Density (μV^2^). PSD values presented in the table were obtained as an average of the three statistical significant scalp positions (F4, O1 and O2). Values with an asterisk (*) are over +2 Standard Deviation.

### Correlation analysis

No correlations were detected between QEEG scores and age, EDSS or cognitive performance indexed by reaction times and percentage of correct responses.

## Discussion

The present study attempted to replicate the effect of validity described in previous studies that used the Posner paradigm [34,35]. The valid condition, that in which the subject's attention is focused on a region of the visual space, results in a faster response (33 ms) compared to the invalid condition, where the focus of attention has to be reoriented toward another region of the visual field.

Regarding the global differences among the groups, reaction time results indicate a poorer execution of the task in RRMS and BMS patients than in controls. Indeed, the BMS group exhibited fewer correct responses than the control group. Both results indicate a certain cognitive deterioration of the MS groups, noticeably more marked in the BMS group.

It is remarkable that the differences between the pathological and the control group are not caused by a *speed- vs accuracy *trade-off. In this sense, MS groups showed impaired performance on both accounts, i.e. slower reaction times in BMS and RRMS, and lower percentage of correct responses (for BMS patients only). This higher degree of cognitive impairment in the BMS group than the RRMS group has been previously observed [40].

The conclusion in our study is that the development of a subclinical cognitive disability not detected by neurological exploration could progress to greater cognitive deterioration. Therefore, it should be possible to assess cognitive status using behavioural techniques and cognitive paradigms.

Regarding the quantitative analysis of the EEG (QEEG), the main result is that, in general, RRMS patients exhibit a larger amplitude for the high bands of the spectrum (beta-2 and gamma) compared to the control and BMS groups in specific regions of the scalp (occipital bilateral and right frontal regions). The rest of the bands (delta, theta, alpha and beta-1) showed no significant difference among the various experimental groups.

This increment for the spectral high bands coincides with other studies where similar increments have been related to psychiatric diseases [25], or in this particular case, to multiple sclerosis [27]. However, the absence of this increment in the background activity in our study suggests that it is more sensitive to calculate spectral variations during the execution of the cognitive task.

Using the same approach, but with the oddball paradigm as the cognitive task, a similar increment in the high bands was found [33]. In this case, a spectral modulation was observed specifically in frontal regions, not in the posterior areas of the scalp. Therefore, it seems that during attentional tasks for diverse sensorial modalities (visual or auditory), it is possible to find different spectral modulations considering the topographical factor. However, a remarkable difference exists between the two experiments about the time at which the window of the PSD was calculated.

In the auditory study, FFT analysis was done after the arrival of the imperative stimulus, with the logical contribution of the evoked potentials related to the stimulus, and hence the possible influence of ERPs on the lower limit of the beta-band values. In the present experiment, calculation of the PSD was carried out prior to the arrival of the imperative stimulus. In this interval, the average in the time domain shows a well-known component called "contingent negative variation" (CNV). The spectral profile of this component is concentrated mainly in the delta range (0.5. 4 Hz) and is unlikely to cause the modulations observed in the beta and gamma ranges. This experiment confirms that the increment observed for high bands of EEG are not due to the contribution of ERPs and should be considered as an abnormal correlate during attentional process in MS patients.

Before we attempt to define the possible explanation for this modulation, it is necessary to discard some alternatives. One possible concern in our case is ocular contamination. However, if ocular artefacts have an effect on frontal electrodes, similar contribution would be expected in both derivations (F3 and F4), which is contrary to what is found (only F4 showed the statistical difference).

Further evidence is that the differences observed in occipital regions must be considered genuine because these are distant from the ocular source, and the intermediate regions (central and parietal) do not exhibit any increment in the high bands.

The increment of high bands could also be caused in different ways by medication. For example, an increment in the beta band has been detected after administration of psychoactive drugs [25]. Other changes in the EEG could be associated to immunomodulatory substances, such as interferon-beta (IFN-beta), which has been referred by other authors [29]. However, it should be remembered that all the recruited patients were free of any kind of medication during their participation in the experiment (at least one month window).

Another possible interpretation for the increment in the beta-2 and gamma bands could be from a higher level of anxiety, a concerned raised by some authors [41]. Although, this phenomenon could appear in some subjects during the recording, it is unlikely that only RRMS patients experience this anxious. This argument could be applied to other variables, such as a different level of motivation or arousal.

Another concern is the possibility that harmonic components (mainly alpha contribution) could be responsible for differences in other spectral components (beta2 and gamma), as suggested by other authors [42,43]. However, differences were not statistically significant among the various groups for the slowest bands in the spectral EEG (delta, theta or alpha).

Finally, it is necessary to discard inference due to muscle activity (we are grateful to one of the referees for this comment). The same argument for the anxiety or motivational level could be employed. Again, it is improbable that only the RRMS patients showed this activity. Two additional analyses (omitted for brevity) were conducted related to this issue. In the first case, to discard the threat from muscle artefact, we analysed the temporal electrodes (T5 and T6) and no increase in beta and gamma bands was detected on them. On the other hand, another analysis was performed to check if the increase of the spectral modulations was present all along the bands or specifically in the high bands (beta and gamma). A relative power analysis of all the spectra indicated that only beta and gamma bands showed an increase for multiple sclerosis patients and the relative power of slow bands was higher in control subjects.

In the individual analysis of QEEG scores, a relevant finding was the presence of abnormal high band activity in RRMS patients and the absence from the BMS and the control group. About 42% of RRMS patients showed abnormal beta-2 activity (over 2 SD), and 37% of these patients for the gamma band.

This result is truly outstanding; although the sensitivity of the technique is modest for the detection of those subjects with RRMS (42%), the probability that a control subject or a BMS patient would be a false positive can be considered null, at least after this preliminary study and with a discrete sample of patients.

Another relevant result that helps in interpreting the high EEG modulations is the absence of any correlation between QEEG scores and cognitive impairment assessed on behavioural grounds. This result agrees with other studies that failed to find a correlation between physiological parameters and cognitive deterioration [30,44]. One possible cause is that the physiological index (QEEG of high bands) and the cognitive process are not related directly. Indeed, the increment of the high bands of the EEG has been associated with diverse psychiatric pathologies [25] where the cognitive deteriorations can be diverse.

If cognitive status is not related to these modulations, what could be the reason for the modulation of high beta bands of EEG in some RRMS subjects and not in the rest of the groups?

One proposal comes from studies that have suggested adaptive cortical functional changes in response to the progression of the disease [45,46]. In these studies, an increment in the activity of brain areas normally devoted to the performance of a given task was found. But, there was also an additional recruitment of areas not activated in healthy people.

The lack of correlation between cognitive impairment and the QEEG scores in the present experiment suggests that the increment of activation (beta2- and gamma) in the cortex could not always be related to the performance of the cognitive task. Indeed, the BMS group showed a higher degree of cognitive impairment and the QEEG scores remained in the normal range. It is possible that there are several mechanisms activated during the MS course. One possibility that has been pointed out [23] could be an adaptive response in the brain to compensate for cognitive impairment, and on the other hand, a non-specific response in the brain (reflected by the increase of high bands of EEG) activating general compensatory mechanisms in response of the progression of the disease, but not strictly related to the cognitive impairment.

Studies have supported that notion that in the first phases of the disease, some processes of cortical reorganization appear [47] with redistribution of ionic channels [48] as lesions arise in certain tissues. These adaptive processes are thought to occur in the early lesions and remain subclinical [49]. Our interpretation of the increment of the high bands of the EEG is that they are electrophysiological correlates of some of these processes occurring specifically in some of the RRMS patients.

But why do these modulations not happen with the rest of the patients with RRMS and in all BMS patients? First, this might be explained by some RRMS patients still being in early phases of the disease, and have not begun the reorganizational processes. Another possibility is that some of these have patients in fact are already drained of some form of "natural reserve", and therefore begin to develop a permanent disability [50] and no high band increment can be observed.

In the case of the BMS patients, perhaps a slow evolution of the disease is not activating the mechanisms of cortical reorganization and in consequence no modulations of EEG are shown. Some form of this "natural reserve" would be activated to compensate for cognitive impairment [23] but some more general cortical plasticity could not be started (failing to show the increase of high bands of the EEG).

Some challenges are opened after these results. First of all, it seems desirable to increase the sample in order to check if these results are consistently enough for MS population. Another important issue in the future would be to correlate these QEEG scores with MRI features (we are grateful to one of the referees about this comment). In the same sense, it would be interesting to check, from the early beginning of the disease, possible "high band profile" to understand the exact meaning of this correlate and the activation of plastic mechanisms. Particularly interesting would be a follow-up study with RRMS patients and the modulations in the high bands related to relapses or the conversion to the SPMS (secondary progressive multiple sclerosis) form.

This interpretation of the results, also suggests the possibility that the increment of high bands of the EEG is not in fact a specific marker for any pathology, but rather an indicator of a natural reaction in the brain before the appearance of lesions or dysfunctions that can be present in diverse pathologies. Regrettably, it is not possible to confirm this hypothesis definitively by the light of our results. More studies would be necessary to confirm this new point of view in the way that we interpret beta and gamma EEG modulations.

## Conclusion

An increment of the high bands of the spectrum appears in the patients with MS although the localization of this modulation was observed in a different topography compared to other similar experiments probably caused by the use of a different sensorial modality. Also, the study has shown that QEEG scores do not correlate with the cognitive impairment, which indicates a relative independence of both variables as has been described in other studies.

Probably, the most relevant result in the present study is that BMS and RRMS patients exhibited different physiological patterns as could be observed with QEEG scores. The possible interpretation of this fact is speculative, but it points out that the increment of the high bands of the EEG could represent the activation of cortical reorganization processes activated in RRMS patients. In the case of BMS patients, these processes would not be activated which could explain why these patients suffer a slow advance and subclinical manifestation of the disease during years of evolution.

The lack of correlation between QEEG scores and cognitive impairment does not mean a complete independence between these two processes. An increment in the high band QEEG scores could be an alerting signal to activate compensatory mechanisms that of course will help in the cognitive performance of the subject.

## Abbreviations

ANOVA: analyses of variance; BMS: benign multiple sclerosis; CR: correct response; DTI: diffusion tensor imaging; EDSS: expanded disability status scale; EEG: electroencephalography; ERPs: event-related potentials; FFT: fast Fourier transformation; MRI: magnetic resonance imaging; Ms: millisecond; MS: multiple sclerosis; PSD: power spectral density; QEEG: quantitative electroencephalogram; RRMS: relapsing-remitting multiple sclerosis; RT: reaction time.

## Competing interests

The authors declare that they have no competing interests.

## Authors' contributions

MV and JJGR, with CG and PD participated in the planning of the study. MV and JJGR participated in the acquisition and execution of the study and performed the data analysis. MV, CG, GI, EV, PD and JJGR contributed to the interpretation of results. The patients were selected by PD, MB and GI. MV drafted the manuscript. The manuscript was subsequently revised by JJGR, CG, EV and PD and GI, and all authors gave final approval.

## Pre-publication history

The pre-publication history for this paper can be accessed here:


